# Antibody repertoire analysis in polygenic autoimmune diseases

**DOI:** 10.1111/imm.12927

**Published:** 2018-04-16

**Authors:** Rachael J. M. Bashford‐Rogers, Kenneth G. C. Smith, David C. Thomas

**Affiliations:** ^1^ Department of Medicine University of Cambridge Cambridge UK

**Keywords:** antibodies, autoantibodies, autoimmunity, B‐cell, B‐cell receptors

## Abstract

High‐throughput sequencing of the DNA/RNA encoding antibody heavy‐ and light‐chains is rapidly transforming the field of adaptive immunity. It can address key questions, including: (i) how the B‐cell repertoire differs in health and disease; and (ii) if it does differ, the point(s) in B‐cell development at which this occurs. The advent of technologies, such as whole‐genome sequencing, offers the chance to link abnormalities in the B‐cell antibody repertoire to specific genomic variants and polymorphisms. Here, we discuss the current research using B‐cell antibody repertoire sequencing in three polygenic autoimmune diseases where there is good evidence for a pathological role for B‐cells, namely systemic lupus erythematosus, multiple sclerosis and rheumatoid arthritis. These autoimmune diseases exhibit significantly skewed B‐cell receptor repertoires compared with healthy controls. Interestingly, some common repertoire defects are shared between diseases, such as elevated IGHV4‐34 gene usage. B‐cell clones have effectively been characterized and tracked between different tissues and blood in autoimmune disease. It has been hypothesized that these differences may signify differences in B‐cell tolerance; however, the mechanisms and implications of these defects are not clear.

## Introduction

B‐cells produce antibodies and are crucial for effective immunity. B‐cell clones selectively expand following antigen recognition by B‐cell receptors (BCRs). BCRs are the membrane‐form of antibodies and are generated through DNA recombination. B‐cells have the potential to recognize a vast array of pathogens, but diversity in the B‐cell repertoire comes at a price, namely that there is a potential for autoreactivity in a subset of B‐cells. Defects in B‐cell development and function can lead to a breakdown of immunological tolerance and therefore autoimmune diseases, which affect approximately 1 in 12 people worldwide.^1^

**Figure 1:**
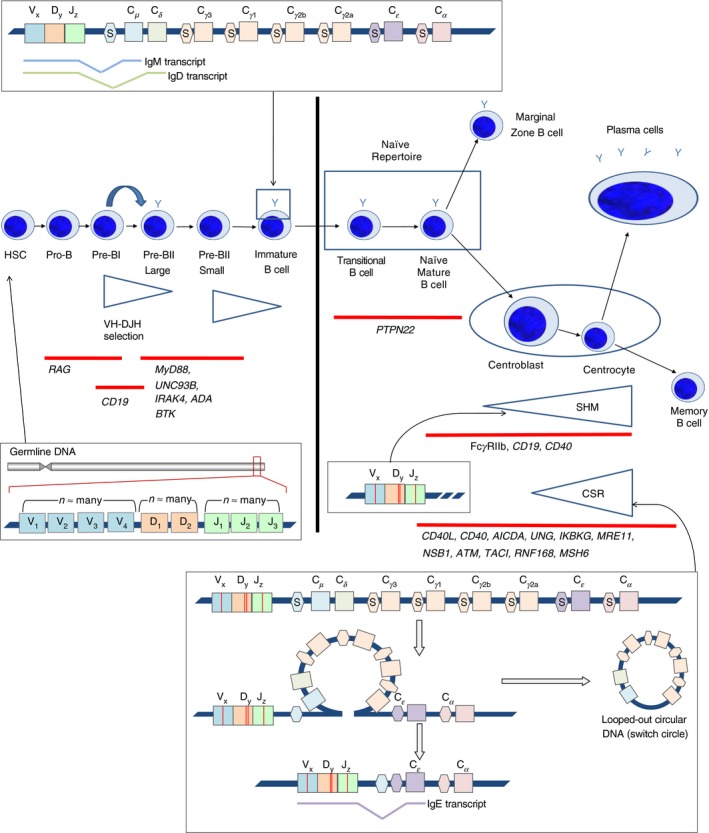
Schematic diagram of the processes of B‐cell differentiation and selection, annotated with known human genetic modifiers of the process and the point in selection at which they are thought to act. B‐cells are generated from haematopoietic stem cells. During B‐cell development, V‐D‐J recombination occurs to produce a functional heavy‐chain. Similarly, V‐J recombination occurs in the light‐chain. The resulting B‐cell receptor (BCR) may be expressed as both surface IgD and IgM on naïve B‐cells through alternative splicing. Somatic hypermutation (SHM) can occur during B‐cell activation, in which mutations are introduced into the V‐(D)‐J region of the BCR. Class‐switch recombination (CSR) can also occur during B‐cell activation, which is a chromosomal deletion process leading to the expression of a different antibody isotype.

B‐cells develop from haematopoietic stem cells and differentiate through several maturation stages in the bone marrow. The germline immunoglobulin heavy‐chain (IgH) gene locus encodes multiple distinct copies of the variable (V), diversity (D) and joining (J) genes, which are separated by over 100 kbp from a much smaller number of DNA segments encoding the constant genes.^2^ During B‐cell development, functional immunoglobulin genes are generated through the deletion of intervening DNA,^3^ creating a IgH gene containing one V, one D and one J gene (VDJ). This encodes the protein sequence for the antigen‐binding region of the IgH protein^2^, ^4^ (Fig. 1). This process of site‐specific recombination is highly orchestrated and mediated by recombination activating genes 1 (RAG1) and 2 (RAG2).^5^, ^6^ The imprecise joining of the V, D and J gene segments leads to the introduction of random deletions and insertions of nucleotides through exonucleases and terminal deoxynucleotidyl transferase (TdT), respectively. This results in sequence diversification at the junctional regions.^7^ Further mechanisms that contribute to the generation of diversity include alternative IgHD reading frames and IgHD–IgHD fusions.^8^ These pre‐B‐cells are selected for functional heavy‐chain by IgV‐D‐J expression and IgH assembly by pairing.^9^, ^10^

Likewise, each IgL chain locus encodes multiple distinct copies of λ chain and κ chain variable (V) gene segments and joining (J) gene segments.^11^ When a cell has successfully rearranged a IgH gene, the B‐cell begins to rearrange the light‐chain genes. Therefore, each mature antigen‐naïve B‐cell typically expresses BCR sequences encoding the heavy‐ and light‐chains. After functional V‐(D)‐J recombination of IgH and IgL chain genes, the resulting immature naïve B‐cells transcribe the IgH and IgL genes, and are able to produce IgD and IgM immunoglobulin isotype by alternative splicing of the transcript to fuse the μ and δ exon to the IgHJ exon, respectively.^12^ At this immature B‐cell stage, the cells are first tested for tolerance to self‐antigens.

### Central tolerance

Immature B‐cells that have no reactivity for self‐antigen leave the bone marrow. For self‐reactive B‐cells, there are four possible fates.

1. Deletion – this predominates when the self‐antigen is multivalent.
2. Receptor editing – the continued expression of RAG means that the light‐chain of any self‐reactive B‐cell can be deleted and replaced with another sequence. Receptor editing is particularly amenable to analysis by BCR sequencing (Box 1).
3. Anergy – a state of unresponsiveness that occurs when B‐cells encounter weakly cross‐linking self‐antigens of low valence.
4. Ignorance – the B‐cell does not encounter the self‐antigen for which it is specific in the bone marrow. It might yet do so in the periphery.

Box 1 **Receptor editing**

If a maturing naïve B‐cell has high affinity for self‐antigens or does not form a functional BCR, the cells may be removed by developmental arrest and/or induced programmed cell death in the bone marrow. These B‐cells can be rescued by modifying the V‐J light‐chain recombination so that the B‐cell receptor no longer recognizes self‐antigens or creates a new functional reading frame.^52^, ^116^ This occurs by the process of secondary rearrangement, where renewed IgHV‐D‐J or light‐chain rearrangement can result in expression of a functional and non‐autoreactive BCR. Receptor editing is again mediated by RAG1/2.^117^, ^118^, ^119^ An upstream V gene recombines with the original V‐D‐J rearrangement to generate a BCR with a new BCR specificity. This occurs between a cryptic recombination signal sequence within the original rearrangement, leaving behind a ~5 nucleotide footprint from the original V gene within the secondary rearranged BCR.^120^ As IgHD‐J recombinations (including junctional regions), known as ‘stem sequences’ are stable in instances of secondary rearrangements,^121^ these can be identified in BCR repertoire sequencing. However, exonucleases and TdT may introduce random deletions and insertions at this new junctional region, which may obscure the V replacement footprint.^122^ Although this can confound inference of secondary rearrangements events from BCR sequencing, secondary rearrangements result in longer CDR3 regions, which is a feature that can be tracked using BCR repertoire sequencing data.

Some autoreactive B‐cells escape central tolerance. In humans, approximately 55%–75% of the early immature B‐cells in humans are autoreactive. This decreases to 40% for bone marrow immature B‐cells and transitional B‐cells, and to approximately 20% for mature naïve B‐cells.^13^, ^14^ Autoreactive B‐cells therefore remain present in the peripheral repertoire, and autoantibodies can be found in healthy humans and mice (reviewed in^15^). Therefore, within a human B‐cell population, there will be a continuum of autoreactivity.

### Diversification of the repertoire in the periphery and peripheral tolerance

Through B‐cell activation through antigen binding by the BCR in addition to auxiliary signals, BCR genes can be further diversified through somatic hypermutation (SHM) in the germinal centre^16^ (Box 2) and/or undergo class‐switch recombination (CSR; Box 3). In some species, gene conversion can also take place. All three processes are dependent on the activation‐induced deaminase (AID) enzyme. SHM diversifies the variable region by introducing point mutations and this leads to affinity maturation. Clearly, the process of SHM also has the potential to drive the formation of newly autoreactive B‐cells in the periphery. For autoreactive cells that escape central tolerance, peripheral tolerance mechanisms can prevent overt autoreactivity. These include: (i) deletion of autoreactive B‐cell clones in the periphery; (ii) the functional suppression of B‐cells through anergy; and (iii) immunomodulation through regulatory T‐cell and B‐cell subsets; and (iv) a lack of T‐cell help from a cognate autoreactive T‐cell.

Box 2 **Somatic hypermutation (SHM)**

SHM is a process that introduces point mutations and, occasionally, insertions and deletions into the variable regions of the heavy‐chain immunoglobulin, where some of the resulting populations are expanded through positive selection for higher affinity antigen binding.^123^ These lead to some B‐cells improving their antigen specificity and affinity to the antigen, often by several orders of magnitude.^124^, ^125^ This process is mediated through the action of cell type‐specific expression activation‐induced cytosine deaminase enzyme (AID). SHM can be determined from BCR sequencing data on comparison with reference germline genes. However, SHM needs to be distinguished from germline alleles present in an individual that are not recorded in a reference database. Germline genes may be inferred from sequencing data (through programmes such as IMPre^126^), against which SHM may be determined.

Box 3 **Class‐switch recombination (CSR)**

There are five major classes of antibody isotype, namely IgM, IgD, IgG1/2/3/4, IgA1/2 and IgE, each with distinct functions through engagement of different activating or inhibitory Fc receptors, and leading to the activation of different immune cells. Through varying the isotype, an antibody can exhibit significant differences in antigen avidity, through dimerization or polymerization of IgA1 and IgM, respectively,^127^, ^128^ as well as binding distinct sets of antibody (Fc) receptors, and ultimately inducing distinct immune responses.^129^ B‐cell activation can lead to isotype class‐switching from IgM to IgG, IgA, IgD or IgE through recombination and deletion processes, known as CSR. This process is coordinated by activation‐induced cytosine deaminase enzyme (AID) and occurs primarily within the germinal centre through close cooperation between B‐cells and T‐helper cells via the interaction of B‐cell surface CD40 protein and CD40L, which is expressed by activated T‐helper cells.^130^

Some individuals will generate and retain a greater number of autoreactive clones or clones with higher affinity for particular self‐antigens arising at any of these stages of diversification. Such individuals may be at higher risk of developing an autoimmune disease.

B‐cell receptor sequencing is helping us address key questions in the field of B‐cell repertoire development, and how B‐cell populations differ between health and disease. B‐cells play important roles in a range of autoimmune diseases. Here, we discuss the current research on B‐cell repertoire in the most well‐studied of these polygenic autoimmune diseases with evidence for the role of B‐cells in pathology, namely systemic lupus erythematosus (SLE), multiple sclerosis (MS) and rheumatoid arthritis (RA).

## SLE

Systemic lupus erythematosus is an autoimmune disease that is characterized by immune complex deposition with concomitant inflammation in almost any organ. Renal involvement, in the form of glomerulonephritis, occurs in about 40% of cases and is associated with a higher mortality. Over 100 autoantibody targets are associated with SLE, and antinuclear antibodies are present in more than 95% of patients. Autoantibodies can be present years before the onset of clinical symptoms.^17^ Transfer of human autoantibodies from patients with SLE can cause glomerulonephritis and proteinuria in murine experimental systems,^18^, ^19^ emphasizing that they can be pathogenic. Genome‐wide association studies (GWAS) have shown that over 80 loci can contribute to the risk of developing SLE.^20^ The pathogenesis of the disease is complex, but several broad themes emerge. Multiple genes involved in: (i) the clearance of immune complexes and apoptotic cells; (ii) the regulation of innate immunity, such as the type 1 interferon and complement pathways; and (iii) the regulation of lymphocyte activation and function have been implicated.

The pathogenicity of autoantibodies and the large number of GWAS risk loci that are involved in B‐cell development and function suggest B‐cells are a key driver of disease.^21^, ^22^ A central question, then, is how the B‐cell repertoire is altered in SLE and whether this can be related to specific polygenic or monogenic drivers of disease. Here we discuss the current studies performed on B‐cell repertoire in SLE (summarized in Table 1).

**Table 1:** BCR repertoire sequencing analyses in SLE

References	Sample number	Peripheral blood B‐cells^a^				Other
		IGHV4 family usage increased	Clonal expansions observed	Shorter CDR3 lengths than healthy controls	Increased SHM than healthy controls	
(Fraser *et al*. 2003)^45^	1 SLE patient	No (GC B‐cells observed only)				Increased IGHV5 family usage and underrepresentation of IGHV1 family usage.
(Arbuckle *et al*. 2003)^17^	8 SLE patients	IGHV4‐34 increased			Mixed	A fraction of antibody‐secreting cell clones contained autoantibodies without mutation.
(Demaison *et al*. 1996)^35^	4 SLE and 5 healthy individuals	IGHV4 family variable dependent on stage of disease				
(Odendahl *et al*. 2000)^23^	6 active SLE, 7 SLE under therapy, and 14 healthy controls	IGHV4 family usage increased in CD27^high^ plasma cells				Preferential usage of IGHV3 family in CD27 + IgD+ memory B‐cells.
(Tipton *et al*. 2015)^36^	8 SLE patients and 8 vaccinated healthy controls	IGHV4‐34 increased in ASCs	Yes: polyclonal expansions		No: lower SHM in ASCs	
(Liu *et al*. 2017)^46^	10 SLE and 6 healthy individuals		Yes	Yes		Significantly higher levels of percentage of charged amino acids, namely arginine, within CDR3 regions than healthy controls.
(Yin *et al*. 2013)^47^	4 SLE and 4 healthy individuals		Yes			
(Dorner *et al*. 1998)^52^	1 untreated SLE patient				Yes	From light‐chain BCR sequencing. Increased receptor editing in SLE also.
(Sfikakis *et al*. 2009)^53^	7 SLE and 4 healthy individuals				Yes	SLE patients exhibited increased levels of SHM.

a Unless otherwise stated.

ASC, Antibody secreting cell; BCR, B‐cell receptor; CDR3, complementarity determining region3; SHM, somatic hypermutation; SLE, systemic lupus erythematosus.

### B‐cell population differences in SLE

Patients with SLE exhibit significantly different B‐cell subpopulation frequencies in peripheral blood. These may be a cause or consequence of autoimmunity, but they should be considered when discussing how the BCR repertoire differs from healthy controls. The abnormalities include the following.

- Significantly decreased numbers of naïve B‐cells.^23^
- More self‐reactive B‐cells than healthy individuals.^24^, ^25^
- Increased frequencies of double‐negative (CD27‐class‐switched) B‐cells,^26^, ^27^, ^28^ which has been shown to correlate with increased autoantibody titres.^26^
- Increased levels of *CD19* ^*low*^ *CD27* ^*high*^ plasma cells,^23^ which may be a consequence of systemic inflammation.^29^

### Germline repertoire variation in the Ig locus in SLE

One reason for studying the BCR repertoire is that variation in germline immunoglobulin heavy‐chain (IGHV) genes has been associated with disease susceptibility. Homozygous deletions of IGHV3‐30*01 and IGHV3‐30‐3 were found to be enriched 2·8‐fold in SLE patients with nephritis compared with ethnically matched healthy individuals, and SLE patients with these deletions exhibited higher titres of anti‐DNA antibodies.^30^, ^31^ This deletion has also been shown to be associated with susceptibility to chronic idiopathic thrombocytopaenic purpura^32^ and Kawasaki disease^33^ (reviewed in Watson *et al*.^34^).

### Peripheral B‐cell repertoire analysis in SLE

Analysis of peripheral blood B‐cells using BCR sequencing can provide important information in four key areas, summarized in Table 1. These are as follows.

- Variable gene usage.
- Clonality and CDR3 length.
- SHM.
- CSR.

#### Variable gene usages in SLE

B‐cell receptor sequencing of antibody repertoires in patients with SLE have shown significant changes in IGHV gene usages compared with healthy individuals. This has been observed in multiple studies, suggesting differences in B‐cell selection and antigen‐specific B‐cell clonal expansion. All the BCR sequencing studies to date have shown an enrichment of IGHV4 gene family usages,^17^, ^23^, ^35^, ^36^ specifically IGHV4‐34.^17^, ^36^ IGHV4‐34 was the dominant serum autoantibody IGHV gene.^17^ Notably, IGHV4‐34 is strongly associated with autoreactivity, with unmutated IgHV4‐34 genes containing the *AVY* motifs in the framework region 1 known to recognize I/i self‐antigen against red blood cell antigens.^37^, ^38^ IGHVH4‐34 gene‐containing antibodies have also been shown to recognize other autoantigens and include anti‐DNA antibodies,^39^, ^40^, ^41^, ^42^ rheumatoid factors (antibodies against the Fc portion of IgG),^43^ as well as commensal bacteria^44^. Some other IGHV families have also been found to be enriched in peripheral blood B‐cells SLE, including IGHV1 and IGHV3.^35^, ^45^ These data are therefore consistent with the idea that the peripheral B‐cell repertoire may be skewed towards autoreactivity in patients with SLE.

#### Clonality and CDR3 region composition of antibodies in SLE

High‐throughput sequencing of BCR repertoires from peripheral blood has shown that patients with SLE exhibit increased B‐cell clonality compared with heathy individuals.^46^, ^47^ This is characterized by polyclonal (multiple) B‐cell expansions.^36^ This is possibly secondary to increased numbers of plasmablasts. In a patient with active SLE, it is likely that plasmablasts generated by the ongoing immune response will be more numerous in peripheral blood. As these plasmablasts have higher levels of BCR RNA per cell, the apparent clonality of the peripheral B‐cell population may increase when sequencing BCR repertoires are sourced from B‐cell RNA.

The complementarity determining region 3 (CDR3) is the most variable region of the antibody sequence (Fig. 1). Longer CDR3 lengths have been associated with both auto‐ and polyreactivity.^48^ Interestingly, patients with SLE display significantly shorter CDR3 lengths in B‐cells from peripheral blood^46^ than controls. Again though, this might be due to increased proportions of plasmablasts in peripheral blood in SLE as naïve B‐cell BCRs tend to have longer CDR3 lengths than antigen‐experienced B‐cells.^49^ Some of the difficulties interpreting such data could be resolved through isotype‐specific BCR sequencing or through investigation of cell‐sorted B‐cell populations, including naïve, memory and plasma cells. As well as changes in CDR3 length, patients with SLE also appear to have qualitative differences in the CDR3 region compared with controls. For instance, CDR3s from B‐cells from patients with SLE code for significantly higher proportions of charged amino acids, such as arginine, but the functional significance of such changes is unclear.

#### SHM in SLE

There are numerous reports suggesting that patients with SLE exhibit increased levels of SHM compared with healthy controls. This provides potential mechanistic insight into the pathogenesis of SLE. If SHM is not stringently controlled and/or B‐cells in the germinal centre receive inappropriate help from autoreactive T‐cells, then autoimmunity might ensue. Accordingly, Dorner and colleagues described increased levels of SHM in SLE from CD19 + B‐cells^23^, ^50^, ^51^ as well as CD27^hi^ plasma cells.^23^ These authors also showed that the peripheral memory BCR repertoire in SLE is shaped by abnormal selection, increased SHM and increased receptor editing.^52^ In agreement with this, Sfikakis et al.^53^ showed increased levels of SHM in SLE.

#### Isotype in SLE

Certain isotypes are associated with autoreactivity, and potentially pathogenicity, in SLE. IgG anti‐dsDNA antibodies have been found to be more closely associated with SLE disease activity and tissue damage than IgM antibodies.^54^ Indeed, some studies suggest that IgM anti‐DNA antibodies may be protective,^55^ whereas other isotypes may also play a role in disease.^56^, ^57^, ^58^ However, there are no systematic BCR sequencing studies in SLE that incorporate analysis of isotypes. Isotype‐resolved BCR repertoire sequencing on peripheral blood or tissue B‐cells subpopulations may be able to provide clues to the extent to which a particular clone has undergone CSR and the relative contributions of each isotype to a certain autoantibody specificity.

#### B‐cell repertoire during or after therapy in SLE

Systemic lupus erythematosus is currently treated with anti‐malarial drugs such as hydroxychloroquine. Some patients are escalated to anti‐proliferative medication such as azathioprine, methotrexate or mycophenolate mofetil in more severe disease. Corticosteroids are frequently used during flares to gain control of disease activity. B‐cell depletion therapies with, for example, rituximab (anti‐CD20) can be used in refractory disease. There is wide variation in the treatment regime used in patients with SLE, and limited data on how the repertoire changes with therapy. An interesting and unresolved question is whether any particular features of the repertoire correlate with either sustained clinical remission or treatment resistance. It is known that certain B‐cell subsets such as antigen‐experienced CD27 + IgD‐class‐switched memory B‐cells are not well targeted by therapy and that this may contribute to treatment resistance. A study of the BCR repertoires of two patients with SLE before and after high‐dose glucocorticoid therapy showed that IGHV3 gene family usage decreased after treatment,^59^ but CDR3 region composition was similar at all time points. While the titre of some anti‐nuclear antibodies reduced after treatment, they did not find evidence of a reduction in clone sizes over this time. In a study of patients with active SLE given rituximab, clonally related B‐cells were found to be persistent in all seven patients, but not found in any of the four age‐matched healthy controls.^53^ This is consistent with preferential depletion of naïve and CD20^high^ B‐cells, with the surviving B‐cell population consisting of CD20^low^ B‐cells, including plasmablasts^60^ and mucosal IgA + plasmablasts that are not removed by B‐cell depletion therapy. As yet, however, there has been no large‐scale analysis of BCR usage in a large number of patients on ‘standard‐of‐care’ non‐B‐cell depleting therapy.

### Conclusions in SLE

These studies have shown that the B‐cell repertoire differs between healthy controls and patients in SLE. However, it is still unclear how these relate to disease pathology, either as a cause or consequence of chronic inflammation. It is possible that both defects in central and peripheral tolerance in inadequate removal of autoreactive B‐cells in SLE and, to some extent, these could be dissected through the study of monogenic drivers of autoimmunity.

A good example of this is a paper by Menard *et al*. They used BCR sequencing and ELISA of recombinant antibodies to study the R620W polymorphism in *PTPN22* that is implicated in susceptibility to autoimmune disease. They showed that B‐cells from carriers of this *PTPN22* risk allele contained high frequencies of autoreactive clones compared with those from non‐carriers showing how a single polymorphism at one genetic locus can affect the B‐cell repertoire.^61^ This *PTPN22* polymorphism is a gain‐of‐function variant leading to reduced B‐ and T‐cell receptor signalling,^62^, ^63^ and has been associated with a range of autoimmune diseases, including RA,^64^, ^65^ type 1 diabetes^66^ and SLE.^67^ Similar studies on variation in other genes are likely to provide further useful information on how specific biological pathways regulate the B‐cell repertoire.

Unanswered BCR repertoire questions remaining in SLE include the following.

- What are the differences in B‐cell repertoire in SLE patients on an isotype level and the differences between peripheral blood and affected tissues?
- How do specific genetic loci affect the B‐cell repertoire?
- How does the repertoire change with disease course and with therapy?
- Can we use BCR sequencing to predict disease prognosis or outcome?

## MS

Multiple sclerosis is an autoimmune disorder characterized by inflammation in the central nervous system (CNS).^68^ It is the most common chronic neurological disorder in young Caucasian adults.^69^ MS initially presents as transient inflammation leading to demyelination of neurons, and is characterized initially by relapsing and remitting episodes of neurological dysfunction. Remyelination with functional recovery may occur between episodes in early disease. Over time, however, the pathology is dominated by extensive microglial activation and neurodegeneration. Functionally this leads to progressive disability.^68^ As with any autoimmune disease, the pathogenesis of MS is driven by a combination of genetic susceptibility and environmental factors.^70^, ^71^, ^72^ The presence of B‐cell clonal expansions and oligoclonal immunoglobulin bands (i.e. an increased concentration of a restricted number of antibodies) in the cerebrospinal fluid (CSF) of the majority of patients suggests a role for B‐cells in MS pathology.^69^ Intrathecal plasma cells, B‐cells, antibody and complement activation have all been observed in MS,^73^ and intrathecal antibody production was noted in very early studies of the disease.^74^

The positive effects of B‐cell depletion therapies and the observation that IGHV2 gene polymorphisms have been associated with susceptibility to MS^75^, ^76^ indicate that B‐cells and antibodies play a significant role in MS. Many autoantigens have been proposed as pathogenic antibody targets in MS, including those derived from the following.

- Myelin‐derived antigens [such as myelin oligodendrocyte glycoprotein (MOG), myelin basic protein (MBP),^77^ proteolipid protein and myelin‐associated glycoprotein].
- Oligodendrocytes (such as CNPase, transaldolase and transketolase).
- Axons (such as neurofilament light‐chain, tubulin and neurofascin).
- Astrocytes (such as potassium channel KIR4.1).
- Ubiquitous autoantigens (such as heat‐shock proteins and nuclear proteins).
- Microbial antigens.^77^, ^78^

Given the evidence that B‐cells contribute to de‐myelination, there have been numerous studies of the B‐cell repertoire in MS, typically focussing on B‐cells in CSF and blood (summarised in Table 2). A particularly exciting aspect of repertoire analysis in MS is the ability to compare the repertoire of B‐cells that have infiltrated the CNS with those in the peripheral blood. This may give vital clues to disease pathogenesis and how the immune response at the site of inflammation differs from the systemic response.

**Table 2:** BCR repertoire sequencing analyses in MS

References	Sample number	CSF infiltrating B‐cells				
		IGHV4 family usage increased	Clonal expansions observed	Clones spanning CSF and peripheral blood	B‐cells undergone SHM	B‐cells undergone CSR
(Baranzini *et al*. 1999)^82^	10 MS and 4 non‐MS controls	Yes: increased IGHV1‐69, IGHV4‐34 and IGHV4‐39				
(Qin *et al*. 2003)^69^	16 MS patients and 32 with other neurological diseases	Yes, as well as IGHV3 and IGHV1	Yes		Yes, and high replacement‐to‐silent ratios	
(Palanichamy *et al*. 2014)^83^	8 MS patients	Yes		Yes		
(Beltran *et al*. 2014)^84^	12 MS patients, 7 patients with other neurological diseases, and 8 healthy control subjects.	Yes		Yes	Yes	Yes
(Stern *et al*. 2014)^79^	5 MS patients		Yes	Yes	Yes	Yes
(Eggers *et al*. 2017)^80^	39 MS patients		Yes, diversity correlated with B‐cell infiltration.	Yes	Yes	
(Colombo *et al*. 2000)^85^	10 MS patients and 10 patients with other neurological disorders	Yes, as well as IGHV3	Yes: oligoclonal bands from PCR amplification products.		Yes	
(Lomakin *et al*. 2014)^131^	8 MS patients	Yes (after selection of B‐cells reactive against MBP, Epstein–Barr virus LMP1 and MOG)				

CSF, cerebral spinal fluid; CSR, class‐switch recombination; LMP1, latent membrane protein 1; MBP, myelin basic protein; MOG, myelin oligodendrocyte glycoprotein; MS, multiple sclerosis; PCR, polymerase chain reaction; SHM somatic hypermutation.

### B‐cell repertoire analysis in MS

As before, however, the studies can be divided into those providing insight into variation in the following.

- Variable gene usage.
- Clonal expansion.
- SHM.
- CSR.

#### Variable gene usage in MS

The presence of oligoclonal immunoglobulin bands in the majority of patients with MS suggests that B‐cell clonal expansion is a feature of the disease,^69^, ^79^, ^80^ specifically as IgG and IgM.^81^ However, there is no definitive evidence that these B‐cells target myelin or other CNS components. Through both spectrotyping and high‐throughput sequencing, increased usage of IGHV1 and IGHV4 families,^69^, ^82^, ^83^, ^84^ specifically IGHV1‐69, IGHV4‐34 and IGHV4‐39, have all been found in the brains of patients with MS.^82^ Overrepresentation of IGHD2, IGHD3 and IGHJ4 families has also been described.^82^ More recent studies using high‐throughput sequencing have shown B‐cells in the CSF of patients with MS are not representative of peripheral blood lymphocytes, indicating tissue‐specific clonal B‐cell expansion and localization.^85^

The composition of the BCR repertoire was found to be distinct between peripheral blood and CSF, with markedly different frequencies of IGHV, D and J usages.^79^ Compared with the periphery, the IGHV4 family was found to be overrepresented in the CNS of matched CSF samples, suggestive of enrichment of antigen‐specific B‐cells into this region. The difference between peripheral and CSF compartments may alternatively be due to differences in B‐cell subpopulations: the majority of B‐cells in MS patients in CSF have memory or short‐lived plasmablast phenotypes,^86^ which have been shown to have a distinct IGHV gene usage profile to that of naïve B‐cells, which comprise the majority of peripheral blood B‐cells.^87^

#### Clonal expansion, SHM and CSR in MS

B‐cell receptor sequencing has shown that CSF‐infiltrating B‐cells exhibit evidence of somatic mutation^69^, ^79^, ^80^, ^84^, ^85^, ^88^ and CSR.^79^, ^80^, ^83^, ^84^ Indeed, ectopic lymphoid tissues have been found in the meninges of patients with MS and these exhibit germinal centre activity, and both IgM and class‐switched B‐cells have been found in the CSF. Beltran and colleagues found that IgM+ B‐cells from CSF showed high degrees of SHM, whereas IgM+ B‐cells from peripheral blood were primarily unmutated because they were naïve B‐cells.^84^

Somatic hypermutation analysis may also be used to estimate the evolutionary relationship between B‐cells of different phenotypes or from different anatomical locations. This allows direct inference of clonal tracking between peripheral blood and CSF. Indeed, clonally related B‐cells have been identified spanning both the peripheral blood and CSF in several studies derived from post‐germinal centre B‐cells, as evidenced by extensive levels of SHM.^80^, ^83^ These studies are suggestive of bi‐directional exchange of B‐cells across the blood–brain barrier. Interestingly, founding members of B‐cell clones, defined as B‐cells expressing BCRs most closely resembling germline, were more often found in draining cervical lymph nodes (CLNs), whereas more mature clone members, defined as B‐cells expressing hypermutated BCRs, were observed in both draining CLNs and the CNS itself.^79^ However, there is still controversy about where SHM and class‐switching occurs in MS. For instance, Stern *et al*. suggest that the majority of B‐cell maturation occurs outside the CNS in secondary lymphoid tissue.^79^ By contrast, Palanichamy *et al*. suggest that SHM may occur in both the CNS tertiary lymphoid structures and in the CLN.^83^ Indeed, a third study has shown that B‐cell lineages with members in both the CNS and CLN are prone to undergo additional rounds of affinity maturation,^86^ further suggesting ongoing and complex B‐cell dynamics between sites of affinity maturation, circulation and lesions.^86^ The differences in conclusions from these studies are likely to result from differences in sampling sites and depth. There is also likely to be significant variation between patients, including variation in their disease course or treatment.

#### B‐cell repertoire during or after therapy in MS

There are few studies on the effect of treatment on antibody repertoire in MS. A study of three patients with CNS demyelination after a single dose of rituximab corresponded with significant loss of peripheral B‐cells, including IgG memory B‐cells.^89^ Within the IgG memory B‐cell population, there was no significant change in IGHV, D or J gene usage, CDR3 region length or charge before and after therapy. However, the frequencies of clonally expanded IgG memory B‐cells significantly increased after therapy, corresponding to preferential depletion of naïve and CD20^high^ B‐cells, with the remainder low‐level B‐cell population consisting of CD20^low^ B‐cells, including plasmablasts.^60^ However, further studies are required to determine the differences between therapies and if there are B‐cell repertoire features associated with remission and long‐term outcome.

### Conclusions in MS

Whilst BCR sequencing has highlighted the potential clonal relationships between blood and brain in MS, it is still unknown how and where such B‐cells differentiate and their contribution to pathogenesis. The emergence of high‐throughput screening technologies alongside BCR sequencing may help elucidate the antigenic targets of CSF‐infiltrating B‐cells. However, determining whether B‐cell infiltration is a result of reactivity against a common self‐antigen or a result of infection or bystander activation is, as yet, unclear. Unanswered BCR repertoire questions remaining in MS are as follows.

- What are the differences in B‐cell repertoire in MS between CSF and other (non‐peripheral blood) tissue sites? A key question is the exact location where autoreactive clones arise and proliferate in MS.
- Can BCR sequencing be used to delineate whether there are differences in central and peripheral B‐cell selection in MS patients compared with healthy individuals?

## RA

Rheumatoid arthritis is one of the most common chronic inflammatory disorders mainly targeting synovial membrane of diarthrodial joints, but other systemic manifestations may also present in patients.^90^ Again, the efficacy of B‐cell depletion therapies, such as anti‐CD20,^91^ have pointed to a key role for B‐cells. Alterations in B‐cell tolerance have been posited to play a role in RA. In particular, BCR editing,^92^ clonal deletion and anergy have been shown to be defective.^93^

The pathological nature of autoantibodies in RA remains controversial. Autoantibody production in patients with RA is well described. Rheumatoid factor and anti‐citrullinated protein antibodies (ACPA)^94^, ^95^ are the most well studied. Infiltrating lymphocytes have been identified in the majority of synovial tissue samples in patients with RA.^96^, ^97^ In about 10% of patients, these manifest as large follicle‐like structures, known as synovial germinal centres.^98^, ^99^ Furthermore, studies have shown that the proportion of peripheral blood plasmablasts is positively correlated with disease activity in RA,^94^, ^100^ and these have been found to produce ACPAs.^94^, ^95^ In a manner redolent of SLE and MS, IGHV gene polymorphisms associate with susceptibility to RA, most notably in IGHV1‐69.^101^, ^102^ Given the association of B‐cells in RA pathology, we review the current research in BCR repertoire sequencing in RA (summarized in Table 3). The majority of studies in RA have focused on synovial tissue and peripheral blood repertoires.

**Table 3:** BCR repertoire sequencing analyses in RA

References	Sample number	Synovial B‐cells				
		IGHV4 family usage increased	Clonal expansions observed	Longer CDR3 regions	B‐cells undergone SHM	B‐cells undergone CSR
(Voswinkel *et al*. 1997)^103^	3 RA patients	Yes				
(Pascual and Capra 1992)^104^	Monoclonal antibodies from various diseases	Yes: IGHV4–34				
(Doorenspleet *et al*. 2014)^105^	12 RA patients	Yes: IGHV4–34	Yes. Peripheral blood dominant clones disappeared during active disease and appeared in the synovial tissue	Yes		Yes
(Samuels *et al*. 2005)^92^	9 RA patients		Yes	Yes		
(Lee *et al*. 1992)^106^	1 RA patient	NA: IGK2 light‐chains enriched		Yes	Yes	
(Tak *et al*. 2017)^108^	21 individuals at risk for RA		Yes. Peripheral blood dominant clones disappeared during active disease and appeared in the synovial tissue			
(Kim *et al*. 1999)^99^	1 RA patient		Yes		Some SHM observed.	
(Morbach *et al*. 2011)^110^	31 juvenile idiopathic arthritis patients				Yes, and CSR	
(Amara *et al*. 2013)^109^	6 RA patients				Yes	
(Tan *et al*. 2014)^94^	16 RA patients		Yes			

CDR3, complementarity determining region 3; CSR, class‐switch recombination; RA, rheumatoid arthritis; SHM somatic hypermutation.

### B‐cell repertoire analysis in RA

#### Variable gene usages in RA

Multiple studies have shown enrichment of IGHV4 genes in the BCR repertoires from synovial tissue from RA patients, specifically IGHV4‐34.^103^, ^104^, ^105^, ^106^ This includes synovial plasma cells,^103^ which are antibody‐secreting cells. Studies of the light‐chain BCR repertoire have also shown enrichment of the kappa IGK2 gene family segments in RA synovium B‐cells,^106^ which have been associated with anti‐RF activity.^107^ In agreement with this, Samuels *et al*.^92^ showed evidence of different levels of receptor editing during central tolerance through analysis of kappa light‐chain BCR repertoire. These data are consistent with, though not diagnostic of, a breakdown of B‐cell tolerance in RA.

#### BCR clonality and CDR3 lengths in RA

Several papers have shown evidence of B‐cell clonal expansions in the synovial tissue of patients with RA.^92^, ^94^, ^99^, ^105^, ^108^ Indeed, in a prospective study of 21 individuals at risk of RA, the risk of developing RA during follow‐up was significantly associated with the presence of ≥ 5 dominant BCR clones, defined as clonally related BCRs representing > 0·5% of the total repertoire.^108^ This was validated in an independent prospective cohort of 50 at‐risk individuals. Interestingly, when individuals developed RA, the clones seen in peripheral blood were no longer detectable there but could be found in synovial tissue, suggesting that activated B‐cell clones migrate to target tissue during active disease. This is in agreement with a previous study showing that during active RA, there were multiple dominant clones within the inflamed synovial tissue that were absent from peripheral blood.^105^ Furthermore, within individual patients, the same dominant B‐cell clones were observed in different joints, suggesting that they migrated between sites of inflammation.^108^ Together, this highlights that B‐cell expansions are present at the sites of inflammation in RA; however, the role and antigenic specificities of these expanded clones is unknown.

Interestingly, expanded or dominant synovial B‐cell clones also showed features of autoreactivity. B‐cell clones from patients with RA were enriched for longer CDR3 lengths in both the heavy‐chain^92^, ^105^, ^106^ and light‐chain (kappa, Igκ).^92^, ^106^ B‐cells with BCRs containing long Igκ CDR3 regions (of ≥ 11 amino acids) were found to be autoreactive or polyreactive.^92^ Thus, a picture emerges in which B‐cells from patients with RA and particularly B‐cells from inflamed joints appear enriched for V gene usage that is associated with specificity for self‐antigen.

#### SHM analysis in RA

There is mounting evidence for antigen‐driven B‐cell affinity maturation and selection in patients with RA, particularly within joints. Using single‐cell sequencing and monoclonal antibody expression of B‐cells from the joints of ACPA+ RA patients with active disease, 25% of synovial IgG‐expressing B‐cells were specific for citrullinated autoantigens; however, these were not found in ACPA‐negative RA patients.^109^ While some of the ACPAs bound more than one citrullinated antigen, none was reactive to non‐citrullinated antigen. Furthermore, when SHMs from ACPAs were reverted back to corresponding germline sequences, anti‐citrullinated peptide reactivity was lost.

Despite evidence for SHM within synovial tissue CD20 + B‐cells in RA,^99^, ^105^ the question of whether continued SHM occurs here has not been comprehensively addressed in RA. In a related disease, juvenile idiopathic arthritis, CD27 + IgD‐ and CD27 – IgD‐ B‐cells accumulate in the joints of patients, and express somatically hypermutated and class‐switched BCRs.^110^ Indeed, these cells displayed activated phenotypes, expressing co‐stimulatory molecules CD80/CD86 and were able to activate allogeneic T‐cells more potently than their peripheral blood B‐cell counterparts, consistent with a role in driving disease.

#### CSR in RA

Class‐switched mature memory subsets were enriched in the synovial compartment compared with peripheral blood.^111^, ^112^, ^113^ Switching occurred primarily to IgG and IgA.^114^ Evidence for class‐switched autoreactive B‐cells in RA is also supported by other studies,^105^, ^109^ where ~25% of synovial IgG+ B‐cells were reactive to citrullinated autoantigens in ACPA‐positive RA patients,^109^ and production of ACPAs by RA peripheral blood IgG+ plasmablasts was found in ACPA+ RA patients, but not ACPA− RA or psoriatic arthritis patients.^94^ Tan *et al*. examined B‐cell specificities in RA using DNA barcoding and sequencing of heavy‐ and light‐chains of antibodies expressed by plasmablasts. The antigen‐specificities of antibodies encoded by IgG+ plasmablasts from patients with RA were tested using microarrays from recombinantly expressed antibodies.^94^ They showed that representative antibodies from patients with RA were able to bind cyclic citrullinated peptides from epitopes on *α*‐enolase, citrullinated fibrinogen and citrullinated histone H2B in an ongoing B‐cell response in RA. Furthermore, these antibodies were not reactive to the non‐citrullinated form of these peptides, suggesting that the activated B‐cells that are present during active disease are selected for their ability to bind citrullinated antigens. This demonstrates that class‐switched B‐cells within the synovium are both autoreactive, class‐switched and somatically hypermutated, suggesting a breakdown of peripheral tolerance. However, the sites of SHM and CSR for autoreactive B‐cells are currently unknown.

#### B‐cell repertoire during or after therapy in RA

There are only a small number of studies examining the effect of therapy on the BCR repertoire in RA. Rouziere *et al*.^115^ evaluated the effect of rituximab on B‐cell repertoires in two patients with active RA, and showed that B‐cell depletion lasted between 5 and 7 months. B‐cell reconstitution was characterized by a diverse BCR repertoire and IGHV gene usages similar to that of healthy adults. However, during the early phase of B‐cell reconstitution (5–7 months post‐rituximab initiation) there was an expansion and circulation of B‐cells containing BCRs with significantly higher levels of somatic mutations. This corresponds to preferential depletion of naïve and CD20^high^ B‐cells,^60^, ^111^ and similar to the effects observed in SLE^53^ and MS.^89^ Indeed, it was shown that IgG serum concentrations were significantly reduced after a first infusion of rituximab in a study of 35 patients with RA, but IgA and IgM serum concentrations were stable until 3‐12 months afterwards.^111^ Consistent with this, there was a different IGHV gene usage distribution at this time point, and the B‐cell repertoire was enriched for class‐switched BCRs.^115^ Whilst this demonstrates significant remodelling of the B‐cell repertoire after B‐cell depletion therapy, the level of clonal persistence and association with disease outcome has not yet been assessed.

### Key outstanding questions in RA

Most studies to date have focused on synovium B‐cell population BCR repertoires, highlighting differences from peripheral blood. It is unsurprising that there are significant differences in BCR repertoire between these compartments. However, the most exciting results arise from the presence of dominant clonal expansions in the peripheral blood that precede clinical presentation and therefore might be predictive of disease development. This is especially pertinent as such clones migrate to the synovium in active disease. The next steps will be to determine the sites of autoreactive B‐cell generation in RA: whether these clonal expansions arise within synovial germinal centres, or within other anatomic sites and undergo migration to the synovium. Indeed, sampling of different anatomic sites and B‐cell clonal tracking through the BCR in high‐risk or early clinical RA patients may shed more light on the early dynamics of autoreactive B‐cells.

## Concluding remarks

B‐cell receptor repertoire sequencing has, so far, been used most effectively to characterize and track B‐cell clones between different tissues and blood. Such studies suggest migration of B‐cells between peripheral blood and the CSF in MS, and between blood and synovium in RA.

Often, these autoreactive B‐cells have also undergone SHM and/or CSR, suggesting a role for the germinal centre in their generation. However, the exact location where affinity maturation occurs is not always clear and is an important question. Indeed, the presence of ectopic germinal centres in synovial tissue or CSF in RA and MS, respectively, still allows for the possibility that B‐cell clones matured in one site cannot then migrate to another. In agreement with this, the circulation of members of B‐cell clones present in the inflamed tissues in MS and RA demonstrates that these autoreactive B‐cells are able to undergo systematic immunosurveillance.

A second key finding across these diseases is the distinct patterns of variable gene usages and CDR3 lengths between healthy individuals and patients with SLE, MS or RA. It has been hypothesized that these differences may signify differences in B‐cell tolerance. However, different B‐cell subpopulations exhibit significantly different variable gene usages and CDR3 lengths. Some of these disease‐specific differences may therefore reflect changes in the relative size of B‐cell subpopulations.

The relative contributions of central and peripheral tolerance in developing autoreactive B‐cell clones in these diseases are currently unknown. Autoreactive B‐cell clones clearly arise from a breakdown in tolerance, though whether this arises at the level for central or peripheral tolerance is unknown. BCR repertoire sequencing of sorted naïve and antigen‐experienced B‐cell populations may provide some insight into central tolerance mechanisms in different diseases. It has been argued that defective central tolerance would generate a naïve B‐cell population with a higher proportion of B‐cells with higher affinities to autoantigen. A difference in naïve B‐cell populations could be assessed by skewed V gene usages and CDR3 lengths as well as assessment of affinity for auto‐antigen affinity ELISA. Dissecting the role of peripheral tolerance may be more complex, particularly when central tolerance may also be defective. Differences in the levels and patterns of SHM and CSR have been demonstrated most clearly between SLE and healthy individuals. However, whether this is a result of abnormal naïve B‐cell repertoire generation is not clear.

Much can be learnt about these processes from investigations into Mendelian disorders of B‐cell development. Indeed, the study of the effect of monogenic drivers or GWAS associations of autoimmune disease may help to unravel the role of different stages of B‐cell selection. This was demonstrated by the defective counter‐selection of autoreactive B‐cells in individuals with the *PTPN22* gene polymorphism. The advent of exome and whole‐genome sequencing of individuals with inherited disorders of B‐cell development and function is set to pave the way to identify hitherto unknown coding and non‐coding variation that influences the B‐cell development (and thus antibody) repertoire (Fig. 1).^61^ Indeed, with distinct genetic signals from each autoimmune disease, we are likely to observe distinct differences between different autoimmune diseases.

As presented here, most of the studies to date on BCR sequencing in autoimmune diseases are limited by small numbers of patients. A meta‐analysis of published data is not possible due to the different BCR sequencing methods or sample sources used. The combined approaches of isotype‐specific BCR sequencing coupled with cell sorting of specific B‐cell subsets and other complementary technologies are likely to make great advances in the field. Furthermore, (i) understanding the changes in B‐cell populations over disease course, (ii) associations with disease severity, and (iii) how this responds to different immune‐modulatory therapies will be critical to understand how to improve patient outcomes. While the advances that have been made in treating autoimmune disease have been substantial, many patients experience treatment‐resistant disease despite the armoury of immunosuppressants now available. Similarly, many currently used immunomodulatory drugs have pleiotropic effects and an extensive side‐effect profile. BCR sequencing in the context of autoimmune diseases is therefore both timely and essential.

## Disclosures

The authors declare no completing interests.
